# Comparative chemical genomic profiling across plant-based hydrolysate toxins reveals widespread antagonism in fitness contributions

**DOI:** 10.1093/femsyr/foac036

**Published:** 2022-07-26

**Authors:** Elena Vanacloig-Pedros, Kaitlin J Fisher, Lisa Liu, Derek J Debrauske, Megan K M Young, Michael Place, Chris Todd Hittinger, Trey K Sato, Audrey P Gasch

**Affiliations:** DOE Great Lakes Bioenergy Research Center, University of Wisconsin-Madison, 53726, Madison, WI, United States; Laboratory of Genetics, University of Wisconsin-Madison, 53706, Madison, WI, United States; Center for Genomic Science Innovation, University of Wisconsin-Madison, 53706, Madison, WI, United States; J.F. Crow Institute for the Study of Evolution, 53706, Madison, WI, United States; DOE Great Lakes Bioenergy Research Center, University of Wisconsin-Madison, 53726, Madison, WI, United States; DOE Great Lakes Bioenergy Research Center, University of Wisconsin-Madison, 53726, Madison, WI, United States; DOE Great Lakes Bioenergy Research Center, University of Wisconsin-Madison, 53726, Madison, WI, United States; DOE Great Lakes Bioenergy Research Center, University of Wisconsin-Madison, 53726, Madison, WI, United States; DOE Great Lakes Bioenergy Research Center, University of Wisconsin-Madison, 53726, Madison, WI, United States; Laboratory of Genetics, University of Wisconsin-Madison, 53706, Madison, WI, United States; Center for Genomic Science Innovation, University of Wisconsin-Madison, 53706, Madison, WI, United States; J.F. Crow Institute for the Study of Evolution, 53706, Madison, WI, United States; DOE Great Lakes Bioenergy Research Center, University of Wisconsin-Madison, 53726, Madison, WI, United States; DOE Great Lakes Bioenergy Research Center, University of Wisconsin-Madison, 53726, Madison, WI, United States; Laboratory of Genetics, University of Wisconsin-Madison, 53706, Madison, WI, United States; Center for Genomic Science Innovation, University of Wisconsin-Madison, 53706, Madison, WI, United States

**Keywords:** chemical genomics, *Saccharomyces cerevisiae*, biofuel, lignocellulose-derived inhibitors, anaerobic growth, antagonism

## Abstract

The budding yeast *Saccharomyces cerevisiae* has been used extensively in fermentative industrial processes, including biofuel production from sustainable plant-based hydrolysates. Myriad toxins and stressors found in hydrolysates inhibit microbial metabolism and product formation. Overcoming these stresses requires mitigation strategies that include strain engineering. To identify shared and divergent mechanisms of toxicity and to implicate gene targets for genetic engineering, we used a chemical genomic approach to study fitness effects across a library of *S. cerevisiae* deletion mutants cultured anaerobically in dozens of individual compounds found in different types of hydrolysates. Relationships in chemical genomic profiles identified classes of toxins that provoked similar cellular responses, spanning inhibitor relationships that were not expected from chemical classification. Our results also revealed widespread antagonistic effects across inhibitors, such that the same gene deletions were beneficial for surviving some toxins but detrimental for others. This work presents a rich dataset relating gene function to chemical compounds, which both expands our understanding of plant-based hydrolysates and provides a useful resource to identify engineering targets.

## Teaser

This work quantifies the fitness contributions of <4000 genes to the growth of yeast in 34 different drugs, chemicals, and toxins found in plant-based material used for sustainable biofuel production.

## Abbreviations

*S. cerevisiae*
: *Saccharomyces cerevisiae*
ERMES
: endoplasmic reticulum-mitochondria encounter structure complex
GET
: Golgi to ER Traffic complex; guided entry of tail-anchored proteins (GET) insertase complex.
DMSO
: Dimethyl sulfoxide
MMS
: Methylmethane sulphonate
BMIM-Cl
: 1-butyl-3-methylimidazolium chloride
EMIM-Cl
: 1-ethyl-3-methylimidazolium chloride
CV
: Crystal violet
NAO
: Nonyl-acridine orange
IBA
: Isobutanol
MBO
: 2-Methyl-3-butyn-2-ol
GVL
: Gamma valerolactone
5-HMF/HMF
: 5-OH-Methylfurfural
IIL
: Imidazolium Ionic Liquid

## Introduction

A major goal for sustainable bioenergy is to use non-edible plant biomass to produce renewable energy fuels and other chemical products by microbial factories. The budding yeast *Saccharomyces cerevisiae* has been used extensively in fermentative industrial processes, including biofuel production from sustainable plant-based hydrolysates (Ekas et al. 2019, Nielsen 2019). Although a promising alternative to fossil fuels, there is still the need to decrease costs through improved efficiency of biomass conversion to useful products (Kumar and Kumar 2017, Ekas et al. 2019). Two main bottlenecks challenge this improvement. First, *Saccharomyces cerevisiae* cannot natively ferment pentoses and oligosaccharides released from deconstructed plant biomass, thereby underutilizing a significant carbon fraction (Kricka et al. 2015, Zhao et al. 2020). Second, toxins found in processed plant biomass are stressful to biofuel microbes, and stress responses mounted by cells redirect resources away from bioproduct formation (Palmqvist and Hahn-Hägerdal 2000, Almeida et al. 2007, Liu 2011, Piotrowski et al. 2014, Cunha et al. 2019, Fletcher and Baetz 2020). Thus, a major goal in sustainable biofuel research is to engineer pentose-consuming microbes that are resilient to toxins derived from lignocellulose and pretreatment processes.

Microbial inhibitors found in plant-based hydrolysates are derived from a number of different sources. One source of inhibitors is the biomass pre-treatment method, which can employ chemically-transformative conditions using acid, heat, ammonia, or solvents (Lau et al. 2009, Singh et al. 2015, Baruah et al. 2018). Emerging technologies include solvents that produce purified sugar streams, including gamma-valerolactone (GVL) (Alonso et al. 2013a, Luterbacher et al. 2014) and imidazolium ionic liquids (IIL) such as [C_2_C_1_im]Cl (also known as 1-ethyl-3-methylimidazolium chloride or EMIM-Cl) (Swatloski et al. 2002, Socha et al. 2014, Hou et al. 2017). Despite solvent recovery methods, residual concentrations of these solvents remain in purified carbohydrate streams at levels that significantly inhibit microbial growth and metabolism (Ouellet et al. 2011). A second class of inhibitors is produced via chemical reactions with the plant biomass (Palmqvist and Hahn-Hägerdal 2000, Klinke et al. 2004, Almeida et al. 2007, Jönsson et al. 2013). The largest set includes phenolic compounds that are released during the breakdown of hemicellulose and lignin and comprise a diverse group of molecules including acids, aldehydes, and ketones (Palmqvist and Hahn-Hägerdal 2000, Klinke et al. 2004, Almeida et al. 2007). In contrast, the furans furfural and 5-hydroxymethylfurfural (5-HMF) are generated during acid pretreatment from the dehydration of pentoses and hexoses, respectively (Palmqvist and Hahn-Hägerdal 2000, Almeida et al. 2007). Furans are found in hydrolysates to varying levels, with high concentrations found in GVL-based hydrolysates (Alonso et al. 2013b, Alonso et al. 2013a). A final class of microbial inhibitors is the metabolic end products themselves, such as ethanol (EtOH), isobutanol (IBA) and other commodity chemicals and biofuels that are toxic at high concentrations (Carmona-Gutierrez et al. 2012, Zhang et al. 2015, Kuroda et al. 2019, Mota et al. 2021). Engineering efforts to increase production therefore require concomitant strategies that increase cellular tolerance to those products.

An added challenge to rational engineering of microbes is that hydrolysate composition can vary substantially from batch to batch, because the suite and concentrations of toxins can be impacted by the pretreatment method but also feedstock growth conditions, seasonal effects, and harvesting characteristics (Klinke et al. 2004, Lau et al. 2009, Chundawat et al. 2010, Bunnell et al. 2013, Jönsson and Martín 2016, Ong et al. 2016, Wehrs et al. 2020). Hydrolysate toxins can also exert combinatorial effects due to interactions among inhibitors. All these features complicate engineering efforts to produce microbial factories customized for specific hydrolysates. A deeper understanding of resistance mechanisms to hydrolysate toxins individually and in combination will be critical to producing flexible sets of strains appropriate for handling a variety of complex hydrolysate stresses.

Several studies have investigated the response to particular hydrolysate toxins, including phenolic compounds (Fletcher and Baetz 2020), ionic liquids (Kumari et al. 2020), or various other classes of compounds (Jönsson and Martín 2016, Kim 2018, Li et al. 2022). Yet much remains unknown about mechanisms of toxicity and how to overcome them, especially for sets of toxins variably found together and under anaerobic conditions, which is preferred for the industrial production of fermentative biofuels. One approach successfully applied to understanding mechanisms of pharmaceutical and other drugs is chemical genomics, in which genes and pathways required to survive specific chemicals are identified via high-throughput interrogation of gene-deletion libraries (Winzeler et al. 1999, Giaever et al. 2002, Hillenmeyer et al. 2008, Enserink 2012, Roemer et al. 2012, Giaever and Nislow 2014). Past chemical genomic screens revealed mechanisms of action from many compounds, implicating molecules as potential therapeutic drugs (Delneri 2010, Ho et al. 2011, Lee et al. 2014, Silberberg et al. 2016) and contributing to our understanding of chemical compounds that impact lignocellulosic biofuel production and other industrial processes (Skerker et al. 2013, Pereira et al. 2014, Dickinson et al. 2016, Bottoms et al. 2018, Xue et al. 2018, Fletcher et al. 2019, Kuroda et al. 2019).

Here, we used chemical genomics in *S. cerevisiae* to understand genes and processes required to survive anaerobic treatment with each of 34 inhibitory chemicals, including solvents used in pre-treatment, toxins generated during hydrolysis of plant material, and biofuel products that stress cells at high levels. The results identified classes of toxins based on chemical genomic profiles, suggested mechanisms of cellular defense, and revealed widespread antagonistic gene-deletion effects. Our results raise important considerations for strain engineering to mitigate variable inhibitor concentrations in different types of hydrolysates.

## Methods

### Strains and growth conditions

*Saccharomyces cerevisiae* strains used in the chemical genomics study belong to the ‘3DeltaAlpha’ drug-sensitive yeast deletion collection of 4309 mutants (*MATα pdr1Δ::natMX; pdr3Δ::KI.URA3; snq2Δ::KI.LEU2)* described in (Andrusiak 2012, Piotrowski et al. 2017). The rationale for using this strain is that it will more sensitively capture compound-specific mechanisms rather than generic effects of multidrug components, mainly non-specific exporters. For chemical genomic analysis, yeast strains were grown in a modified version of synthetic SynH3^−^ medium (‘SynBase’ medium) described in (Zhang et al. 2019). SynBase medium used in this study was prepared identically as SynH3^−^ except for the following changes: acetamide, sodium acetate, and cellobiose were not included, and ammonium sulfate was replaced with 1 g/L monosodium glutamate (MSG, Fisher Scientific) and adjusted to pH 5.0 with HCl. Acetamide was removed because it is only present in AFEX-pretreated biomass hydrolysates. Ammonium sulfate was replaced by MSG to allow for antibiotic selection (ammonium sulfate prevents antibiotic selection in yeast (Cheng et al. 2000)).

YPD medium was prepared as previous described (Sherman 2002). Briefly, liquid and plate-based medium contained 10 g/L yeast extract, 20 g/L peptone (YP) and 20 g/L dextrose (D). In validation experiments, strain BY4741 (*MATa his3Δ1 leu2Δ0 met15Δ0 ura3Δ0*) was used as the wild-type parent. Deletion mutations were performed by integration of polymerase chain reaction (PCR) products generated from *loxP-kanMX-loxP* (pUG6) plasmid template (Güldener et al. 1996) and primers containing 50 bp of homology flanking the targeted gene. PCR products were purified and transformed (Gietz and Schiestl 2007) into the BY4741 parent strain. Targeted gene deletions were confirmed by PCR and Sanger sequencing. For engineering gene deletions with the kanMX selection marker, 200 µg/mL Geneticin (US Biological, Swampscott, MA) was added to YPD. For validation of IIL-sensitive strains, SynBase medium with 25 mM EMIM-Cl or 250 mM LiCl were added.

### Chemical genomic experiment

Concentrations for each inhibitor used for the chemical genomics experiment with the yeast deletion library were determined based on estimated inhibition of ∼30% of growth (IC_30_) in SynBase medium with the inhibitor relative to growth in SynBase medium lacking the inhibitor (Table S1, Supporting Information). Chemical compounds insoluble in water were dissolved in DMSO at 100X concentration so that the final concentration of DMSO in SynBase medium was 1% (v/v). For these IC_30_ experiments, area under the curve (AUC) from OD_600_ measurements recorded every 15 min for 24 h in 96-well plates were used as a measure of growth, with the AUC growth for the wild-type strain ‘3DeltaAlpha’ (*MATα pdr1Δ::natMX; pdr3Δ::KI.URA3; snq2Δ::KI.LEU2*) in SynBase or SynBase + 1% DMSO defined as 100% growth. QUADRIS is a suspension and was highly insoluble in SynBase, making OD_600_ reads inaccurate; thus, two concentrations were arbitrarily selected. Benomyl and MMS concentrations were used as previously published (Piotrowski et al. 2017), 10 ug/mL and 0.01%, respectively.

Chemical genomic experiments were performed as previously described (Piotrowski et al. 2015) with modifications. Briefly, the pooled yeast gene-knockout library was inoculated into 1.5 mL of SynBase or SynBase + 1% DMSO containing individual inhibitors at their defined IC_30_ concentration in 24-well plates (Falcon) at an OD_600_ = 0.1. All 24-well plates contained control samples with SynBase or SynBase + 1% DMSO lacking any inhibitor for paired analysis (see below). Inoculated plates were grown in an anaerobic chamber (Coy Laboratory Products, Inc.), containing 1%–2% H_2_, 4%–5% CO_2_, and 90%–95% N_2_ at 30 °C for 24 h, and then transferred into the identical fresh medium at OD_600 _= 0.1 for another 24 h without shaking. OD_600_ measurements were made at the end of each 24 h period by manually resuspending the cell cultures using a pipettor and measuring with a Spark plate reader (Tecan) in the anaerobic chamber. All cell cultures reached between 6.5 to 10 total cell doublings within the two 24 h growth periods. Cells were then harvested and genomic DNA was extracted (MasterPure Yeast DNA Extraction Kit, Lucigen). Growth of the yeast deletion library in all inhibitory and control conditions were performed in independent biological triplicate. DNA barcodes were amplified using specific index and U2 primers (Sigma-Aldrich) and high-fidelity DNA polymerase (Phusion, Thermo Fisher) as previously described (Piotrowski et al. 2015). Barcodes were sequenced using an Illumina HiSeq Rapid Run platform. Sequencing data are available in the NIH GEO database under accession number GSE186866.

### Chemical genomic data processing and functional analysis

Barcode read counts were calculated from up-tag reads using custom python scripts. Gene deletions with specific fitness contributions were identified using linear models in edgeR version 3.26.8 (Robinson et al. 2010), using TMM normalization and glmQLFit comparing paired treatment to control samples, except with MMS and QUADRIS compounds, which were unpaired. Benjamini and Hochberg correction (Benjamini and Hochberg 1995) was used to calculate the false discovery rate (FDR), taking FDR < 0.05 as significant. Results were presented in heat map figures as the log_2_ of the normalized read counts for inhibitor/control ratio. 3233 genes, which were significant for at least 1 of the 34 inhibitors (FDR < 0.05) were selected for clustering and downstream analysis and are presented in Dataset2_mclust_cdt (Supporting Information). Genes clusters in Fig. 5A were defined using mclust package version 5.4.5 (Scrucca et al. 2016) with k = 50 and model EII. Genes were organized within each mclust cluster by hierarchical clustering (Eisen et al. 1998) and data were visualized with Java Treview (Saldanha 2004). Enriched GO categories for each compound were obtained using hypergeometric tests, taking *p*-value ≤ 10^−4^ as significant (we focused on this stringent p-value cutoff because functional categories are highly overlapping and thus cause over-correction by FDR methods).

### Classifying inhibitors based on global fitness profiles

Pairwise Pearson correlations between compounds were calculated based on the log_2_ values comparing each inhibitor versus control, over the 3233 significant genes described above, and results were hierarchically clustered and visualized with corrplot package in R (Wei and Simko 2021). Inhibitors were manually partitioned into compounds categories in Fig. 3 based on off-diagonal similarities.

### Visualizing genetic basis of pairwise inhibitor antagonism

The antagonistic proportion of the union of significant deletions for each pairwise inhibitor comparison was visualized using the R package pheatmap (Kolde 2018). The shared and unique genetic bases of antagonism with EMIM-Cl and ethanol were visualized using the R package UpSetR (Conway et al. 2017).

### Comparison with prior chemical genomics datasets

We incorporated prior results (Lee et al. 2014), which used a similar chemical genomics approach to investigate fitness responses to over 3000 compounds. To do this, we defined gene sets that are diagnostic of particular types of stress, as follows. Lee et al. defined sets of chemicals with shared fitness profiles, referred to as ‘response signature’ compound sets. We associated a given gene with a given response signature set if that gene was more likely to be significant (z > 3.09) within that set of compounds than expected by chance, based on all significant scores for all chemicals, using the hypergeometric test. We did this separately for positive (i.e. beneficial) log_2_ fitness values and for negative (i.e. deleterious) fitness values for each gene. This in effect defines gene sets diagnostic of benefits (Lee_Positive lists) or defects (Lee_Negative lists) in response to particular stressors. We combined these diagnostic gene sets with gene functional categories including Gene Ontology (GO) functional categories, and scored enrichment of such lists for genes whose deletion is beneficial or deleterious to compounds studied here.

### Clustering of functional enrichments

To organize functional enrichments across drugs as shown in Fig. 5B, we took a clustering approach. For each inhibitor, we scored GO and Lee-category enrichments for genes whose deletion was beneficial or detrimental, through separate analyses. We then took the -log_10_(*P*-value) for each enrichment; enrichments specific to deleterious gene-deletion lists were multiplied by −1, and then the categories were hierarchically clustered with Cluster 3.0 (Eisen et al. 1998), and visualized in Treeview (Saldanha 2004).

### Strain engineering and fitness validation

For validating fitness results from the chemical genomics experiments, deletion mutants were reconstructed in the BY4741 background. Strains were cultured overnight in YPD and diluted to OD_600 _= 0.3 in fresh YPD the next day. When the cells reached log phase growth (∼3–4 h after dilution), cells were harvested, washed with sterile water, and inoculated into the appropriate medium at OD_600 _= 0.1–0.2. Strains were inoculated in 200 µL of medium in wells of a 96-well plate and cell densities were measured with a Tecan Spark stacker housed within the same anaerobic chamber described above to measure OD_600_ for 48 h. The slope of the log-transformed exponential phase of each growth curve was used to calculate doubling times. To control for batch effects in multi-plate assays, mutant doubling times were normalized to their within-batch wild-type control.

## Results

### Overview

**Figure 1.**
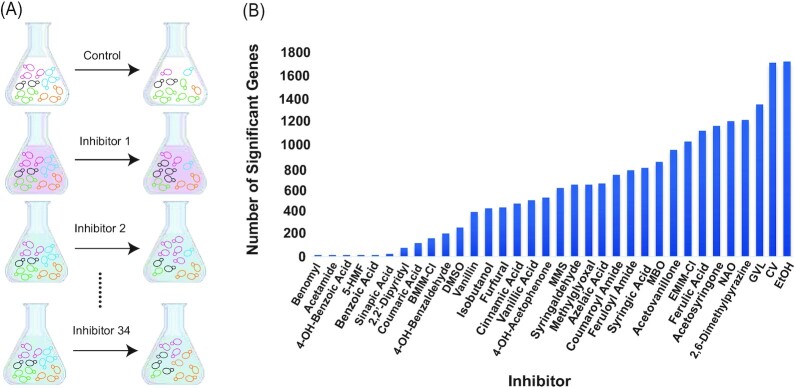
Chemical-genomic analysis. **(A)**Schematic of chemical genomic selections. Each colored strain depicts a different gene deletion strain, before and after exposure to one of 34 different inhibitors. Strain abundance before and after inhibitor exposure is revealed by quantitative sequencing of the strain barcodes. **(B)**The number of gene deletions that significantly influence fitness (FDR < 0.05) for each of the inhibitors, sorted according to the number of affected genes.

We performed chemical genomic selections in a sensitized yeast gene-deletion library (Andrusiak 2012, Piotrowski et al. 2017) to identify genes that impact fitness when cells are grown anaerobically in the presence of individual inhibitors from plant biomass hydrolysates. This library, used in previous studies for profiling of hydrolysates and single inhibitors (Dickinson et al. 2016, Ong et al. 2016, Bottoms et al. 2018), decreases the general multidrug response, and thus allows better identification of genes specific to toxin mechanisms of action. The library includes 4309 strains in which a non-essential gene is replaced with a unique DNA sequence (barcode) flanked by common sequences for barcode amplification. The pooled library of cells was grown anaerobically in synthetic medium (‘SynBase’, see Methods) containing a single inhibitor compound for ∼7–10 generations, alongside a matched control grown in medium lacking inhibitors, in biological triplicate (Fig. 1A, see Methods). We used a variety of compounds found in plant-based hydrolysates, including solvents from pre-treatment methods (such as GVL and the IILs EMIM-Cl and 1-butyl-3-methylimidazolium chloride (BMIM-Cl or [C_4_C_1_im]Cl)), by-products from lignocellulose breakdown (including phenolic compounds, furfural and 5-HMF), and biofuel end-products (ethanol, isobutanol and 2-methyl-3-buten-2-ol (MBO)), as along with several other chemicals, including some with known mechanisms (such as benomyl) (Table S1, Supporting Information). Acetic acid is one of the most prevalent and studied inhibitors in hydrolysates, but it is found at lower concentrations in some pre-treatments of our interest such as AFEX (Chundawat et al. 2010). Thus, we focused more on other inhibitors found in AFEX, GVL, and IIL pre-treated hydrolysates as well as other less studied compounds. The dose of each inhibitor was chosen based on estimated 20%–30% inhibition of growth from dosage curves. Briefly, we compared the growth of the wild type strain with each inhibitor to a control without inhibitors, measuring it every 15 minutes for 24 h (see Methods). Linear modeling of quantitative barcode sequence counts identified genes whose deletion produced reproducible fitness effects in the presence of each inhibitor compared to the paired SynBase medium control (see Methods). Deletion strains that increased in relative population abundance after inhibitor exposure were inferred to experience a fitness benefit, while deletion strains that decreased in abundance were inferred to suffer from fitness defects, thus implicating those genes as important for growing in the presence of that compound. We performed gene ontology (GO) enrichment analysis separately for genes whose deletion is beneficial or detrimental to each toxin. We also compared our results to a prior chemical genomic analysis of over 3000 compounds, including some pharmaceutical drugs and other molecules (Lee et al. 2014). We defined gene sets from that study that are diagnostic of pharmaceutical drugs and other compounds that had been characterized with signature biological responses, and then included those gene sets in our functional enrichment strategy (see Methods). This identified overlap in genes required for model stressors from prior work and compounds studied here, thereby contributing to our understanding of mechanisms of action of hydrolysate toxins.

In total, we identified 3233 gene deletions conferring a fitness effect to at least one compound at a false discovery rate (FDR) of 5%. The number of significant genes ranged from as few as 12 genes for the dose of benomyl used here to 1706 genes affecting ethanol tolerance (Fig. 1B). Although the doses of the chemicals used in the experiment were chosen to impart a consistent level of stress (see Methods), it is possible that some chemical exposures produced more severe stress than others. Another possibility is that the number of significant genes reflects the breadth of the inhibitor effects on cell physiology. For example, genes important for surviving the microtubule disrupting drug benomyl were heavily enriched for genes related to microtubule and tubulin complex assembly (*P* = 1.48e-08, Hypergeometric test, see Dataset1_HyperG, Supporting Information). In contrast, cationic dye crystal violet (CV) and ethanol treatment identified gene lists enriched for a wide range of processes, including transmembrane transport, membrane lipid biosynthesis, and amino acid metabolism in the case of CV, and membrane fluidity, protein unfolding, microtubule integrity, and oxidative stress for ethanol treatment.

### Similarities in fitness profiles reveal classes of inhibitors

**Figure 2.**
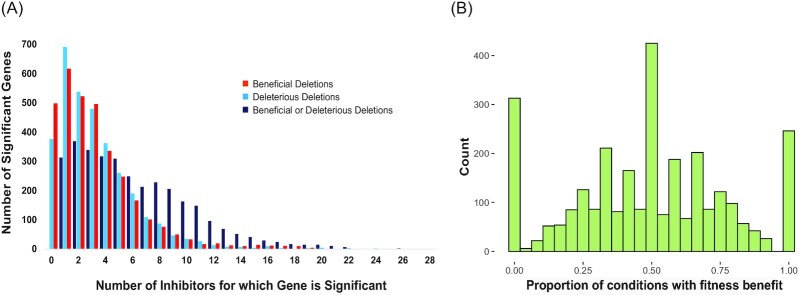
Many gene deletions have an impact on fitness for a large number of inhibitors. **(A)** The number of significant genes is plotted against the number of inhibitors in which their deletion provided a fitness effect, considering inhibitors where the effect was only beneficial (orange), only deleterious (light blue), or either beneficial or deleterious for different inhibitors (dark blue).**(B)** Histogram of the number of gene deletions plotted against the proportion of conditions in which their effect was significant that was beneficial. A minority of genes (559) fall at the tails of the trimodal distribution, indicating that their deletion produces significant fitness effects in only one direction. Most deletions are antagonistic with similar rates of beneficial and deleterious effects across the data.

To better understand the architecture of gene contributions to chemical fitness, we plotted the number of significant gene deletions against the number of compounds in which they had a beneficial or a detrimental effect (Fig. 2A). When considering genes whose deletion had the same effect on fitness across compounds (i.e. only beneficial or only detrimental), most genes were scored as important for only a few compounds. However, plotting the number of significant gene knockouts regardless of the directionality of the fitness effect revealed that a large fraction of genes impacted fitness, in one direction or the other, under many inhibitors. For example, only 32–34 gene deletions produced an exclusive fitness benefit or fitness cost, respectively, for 10 or more compounds; however, when we ignored the directionality of the fitness effect, nearly 200 deletions affected fitness for at least 10 compounds. This result suggests that many gene deletions produce antagonistic fitness effects, *i.e*. a defect in response to one inhibitor but a benefit in response to others.

**Figure 3.**
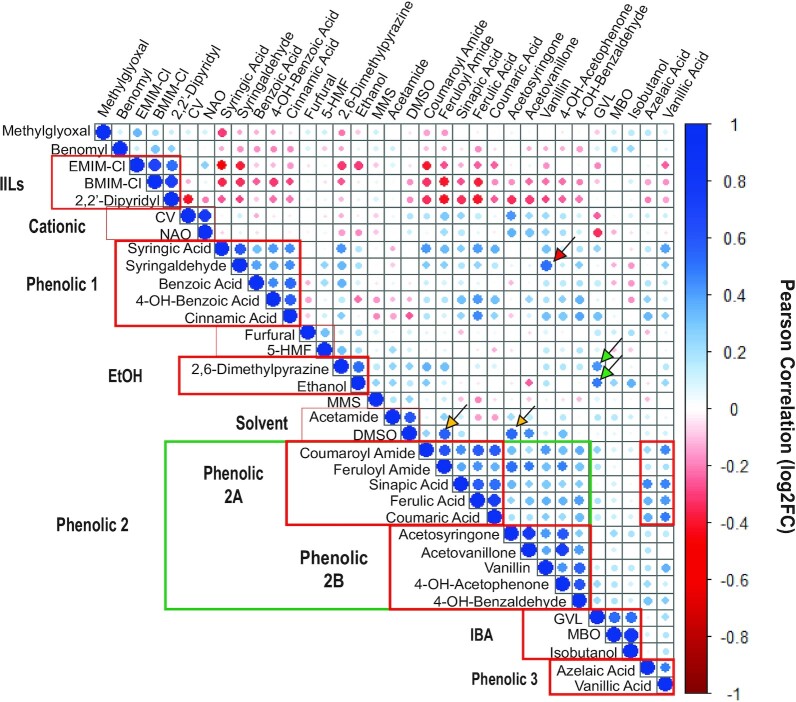
Classification of inhibitors based on similarities in gene-fitness profiles. Inhibitors were classified by hierarchical clustering of the Pearson correlations in log_2_ fitness effects across 3233 genes significant for ≥ 1 compound (FDR < 0.05). Circle size and color is proportional to the correlation in fitness profiles, according to the key. Inhibitor classes were manually defined based on off-diagonal similarities in compound responses (red and green boxes), see text for details. Arrows indicate examples of off-diagonal inhibitor similarities, including specific examples highlighted in the text (red: vanillin to syringaldehyde; green: MBO to ethanol and 2,6-dimethylpyrazine; yellow: DMSO to coumaroyl amide and acetosyringone).

We next classified the inhibitors based on similarities in fitness profiles across all significant genes. We calculated the pairwise Pearson correlation between compounds, based on the log-transformed fitness contributions of 3233 genes whose deletion produced a fitness effect for at least one of 34 inhibitors (Fig. 3). Based on the clustering of the data, we then manually defined boundaries for classes of inhibitors, based on off-diagonal similarities. Some compounds (e.g. methylglyoxal and benomyl) did not correlate well with others and therefore were not grouped into larger classes, but other compounds fell into larger categories. In some cases, these categories related to shared chemical properties of the compounds. In other cases, the groupings were not defined by chemical properties and instead reflected shared cellular response strategies.

In the case of IILs, BMIM-Cl and EMIM-Cl are thought to inflict cellular stress via their cationic components (Stolte et al. 2006, Kumari et al. 2020). However, the global response to these solvents was distinct from cationic dyes CV and nonyl-acridine orange (NAO). Instead, IILs grouped more closely with the metal chelator 2,2′-dipyridyl (see more below). Phenolic compounds were divided into distinct groups that partly correlated with their chemical structures. For example, Phenolic Group 1 included syringic acid (as well as syringaldehyde), benzoic acid, 4-hydroxy benzoic acid, and cinnamic acid. Phenolic Group 2A contained coumaric and ferulic acids and their amide forms, which are prevalent in ammonia fiber expansion (AFEX) pretreated biomass (Keating et al. 2014), along with sinapic acid. Phenolic Group 2B also correlated to these compounds, albeit with lower correlation, and included the ketones acetosyringone, acetovanillone, and 4-hydroxyacetophenone, as well as the aldehydes vanillin and 4-hydroxybenzaldehyde. Phenolic Group 3 included vanillic acid and the non-phenolic azelaic acid; the two compounds also shared similarity in global fitness profiles with compounds in Phenolic Group 2A and, to a lesser extent, other phenolic compounds. In other cases, inhibitor correlations did not recapitulate chemical relatedness. For example, the response to alcohol IBA was more similar to MBO and solvent GVL than it was to ethanol.

Relationships across groups of inhibitors were also informative. In addition to sharing high similarity with IBA and MBO, GVL uniquely shared similarities with ethanol and 2,6-dimethylpyrazine (Fig. 3, green arrows). The latter result is consistent with previous studies showing that GVL and ethanol can damage and permeabilize membranes and produce synergistic effects when in combination (Ding et al. 2009, Huffer et al. 2011, Lam et al. 2014, Bottoms et al. 2018). Interestingly, IILs showed strong cross-group relationships that negatively correlated to the global fitness profile of many other compounds, especially phenolic compounds in Group 2A. This result suggests that many of the antagonistic effects in gene fitness contributions implicated in Fig. 2 emerge due to unique differences in the response to IILs.

### Extensive levels of antagonism in defense mechanisms

The vast majority of gene deletions that affected fitness in more than one condition exhibited antagonism across inhibitors (2362 out of 2921 deletions), and most antagonistic genes were beneficial and deleterious at roughly equal rates across inhibitors (Fig. 2B). Fitness tradeoffs between environments are not unexpected (Qian et al. 2012, Jakobson and Jarosz 2019). However, such tradeoffs could complicate attempts to engineer broadly tolerant yeast strains, because a gene deletion beneficial for surviving one toxin could be highly deleterious for other toxins in the same hydrolysate. Because many of these inhibitors are present in the same hydrolysates, the effect results in widespread antagonistic selectional pleiotropy (Paaby and Rockman 2013), a type of pleiotropy wherein perturbation of a single gene affects multiple components of fitness.

**Figure 4.**
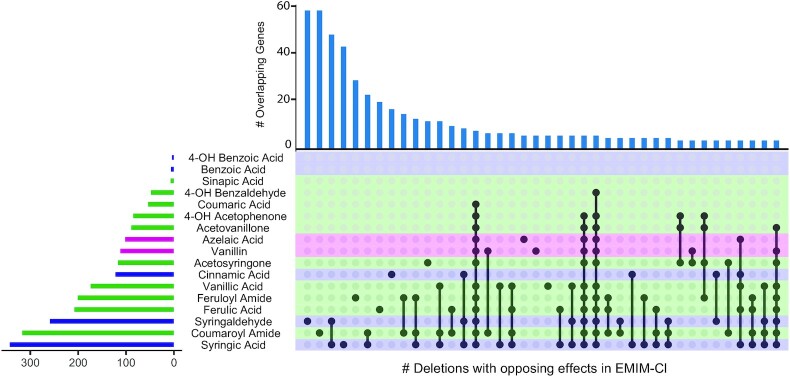
Antagonistic effects with EMIM-Cl are specific to different sets of inhibitors. UpsetR plot shows the number of genes whose deletion presents a fitness change in the opposite direction of EMIM-Cl in response to the indicated subsets of inhibitors. The number of genes in each group is shown in the bar graph at the top, and the number of genes significant for each phenolic compound list is shown in the histogram to the left.

As an orthogonal approach to determine the most relevant instances of antagonistic selectional pleiotropy, we determined the proportion of antagonistic deletions among the union of significant deletions for inhibitor combinations that could be expected to co-occur in real hydrolysates (Fig. S1, Supporting Information). We found evidence of tradeoffs between pre-treatment chemicals, alcohol biofuels, and plant-derived inhibitors. IIL hydrolysates were particularly problematic since IILs demonstrated widespread antagonism with phenolic inhibitors, reinforcing results outlined above. When we examined the identity of genes with opposing effects in EMIM-Cl and individual phenolic inhibitors, we found that there was not a core set of genes responsible for antagonism (Fig. 4). Instead, the genetic basis of antagonism with EMIM-Cl is largely unique for each phenolic inhibitor. An exception to this is the shared set of 48 deletions showing fitness tradeoffs with EMIM-Cl in both syringic acid and syringaldehyde. No genes share opposing effects in EMIM-Cl with all phenolic inhibitors nor with all inhibitors belonging to the same phenolic group.

We also observed high levels of antagonism between IILs and ethanol (Fig. S2, Supporting Information). Indeed, the strongest tradeoff observed was between ethanol and EMIM-Cl, with 28% of gene deletions that were significant to either condition showing antagonistic pleiotropy. Interestingly, the apparent tradeoff between ethanol and EMIM-Cl tolerance did not extend to BMIM-Cl, for which only 5% of deletions had opposing effects in ethanol. Aside from IILs, ethanol also exhibits conflicting cellular responses with a number of lignocellulose-derived toxins, as well as with the two other biofuels included in our screen, IBA and MBO. Other than a group of 95 genes in common between acetosyringone and acetovanillone, the cellular processes underlying antagonism between tolerance to ethanol and other inhibitors again differed amongst inhibitors (Fig. S2, Supporting Information). Taken together, the pervasive antagonistic selectional pleiotropy present in the genetic responses to toxins suggests that optimizing strains to overcome some classes of inhibitors found in hydrolysate could lead to decreased tolerance of others, potentially nullifying any fitness benefits.

### Clustering of gene fitness contributions suggests mechanisms of action

The inhibitor classifications in Fig. 3 were based on global correlations in gene-fitness profiles. However, many compounds have pleiotropic effects on the cell, and thus they may share some aspects of their response with one class of inhibitors but different aspects of their response with others. Therefore, identifying subsets of genes whose deletion similarly impacts subsets of compounds can be especially informative on the chemical's mechanism of inhibition.

**Figure 5.**
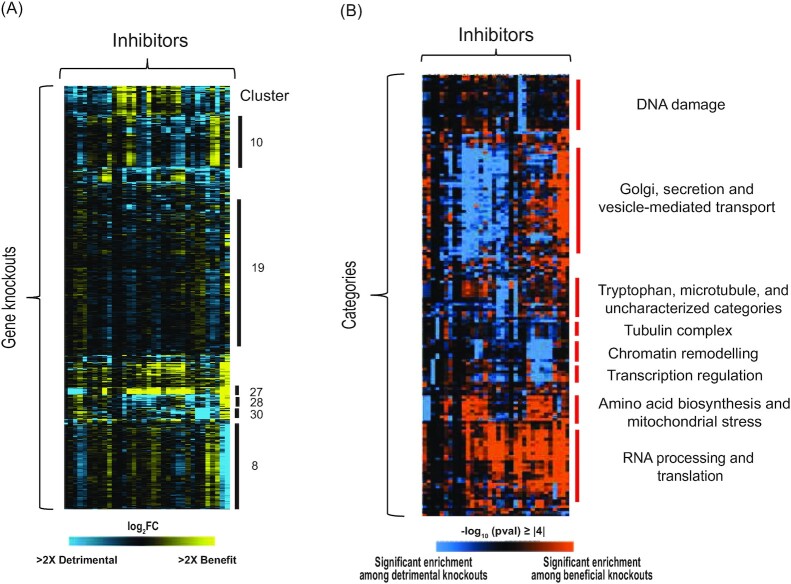
Gene and functional-category clustering inform on inhibitor mechanisms. **(A)** Mclust clustering of 3233 genes whose deletion significantly (FDR < 0.05) influences one or more of 34 inhibitors (Dataset2_mclust_cdt, Supporting Information). log2 fitness effects of gene deletion strains (rows) for each inhibitor (columns) are colorized according to the blue/yellow key. Clusters discussed in the text are annotated to the right. Functional enrichments for clusters can be found in Dataset3_HyperG_mclust (Supporting Information). **(B)** Hierarchical clustering of functional categories as described in the text (Dataset4_HyperG_cdt, Supporting Information). Colors indicate the -log10 (*P*-value) of enrichment for categories (rows) enriched for each inhibitor (columns), with orange indicating enrichment on the inhibitor's beneficial gene-deletion list and blue indicating enrichment on the inhibitor's deleterious gene-deletion list.

We took two approaches to understand similarities and differences in inhibitors responses. First, we used mixture modeling and hierarchical clustering of gene fitness contributions to identify subsets of gene deletions with similar fitness effects across subsets of compounds (Fig. 5A, Dataset2_mclust_cdt (Supporting Information), see Methods). Second, we devised an approach to organize the dense enrichments of functional annotations for each inhibitor's significant gene list, and we looked for similarities in enrichment patterns across subsets of toxins. The utility of this approach is that it can identify toxin relationships based on shared enrichments of the same functional categories, aiding in interpreting cellular responses. For each inhibitor, we converted the enrichment p-values to the negative log_10_ of that *P*-value, which was multiplied by −1 if the enrichment was specific to deleterious gene-deletion lists. We then clustered functional categories based on these scores, which indicate enrichments specific to beneficial gene deletion lists (colorized orange, Fig. 5B) or to detrimental deletion lists (blue, Fig. 5B) separately. A key feature of our approach is the inclusion of pharmaceutical drug and stress-response categories from (Lee et al. 2014) to aid in mechanistic interpretation. We used this analysis to understand physiological mechanisms of several inhibitor classes, as described below.

### Imidazolium Ionic Liquids share similarities and distinctions with other cationic toxins

While IILs are promising solvents for deconstruction of plant biomass, they are well known for their toxicity to *S. cerevisiae* (Ouellet et al. 2011, Dickinson et al. 2016, Higgins et al. 2018). IIL toxicity is linked to their cationic components and their lipophilicity (Stolte et al. 2006, Kumari et al. 2020); while previous work indicated that IILs impact mitochondrial function (Dickinson et al. 2016), their specific mechanisms of toxicity are not completely understood.

Several responses were shared between IILs and cationic dyes CV and NAO, thereby implicating cationic stress. One group of 645 genes (Fig. 5A, Cluster 8) was important for tolerance of these compounds and included genes involved in cation transport such as zinc, iron, and potassium (*MSC2, TRK2, MMT1, FSF1, MMT2, FET5, ARN2, FRE2, SMF3, FRE7, FSF1*, *VNX1, YKE4*). This group also included *SGE1* and *ILT1*, which encode membrane-bound proteins previously implicated in EMIM-Cl tolerance and proposed to export cationic toxins out of the cytoplasm (Higgins et al. 2018). Additional genes in Cluster 8 included *SAT4/HAL4* and *HAL5*, which encode homologous protein kinases that regulate the Trk1/2 potassium transporters (Mulet et al. 1999), hinting that the Trk system may play a role in IIL tolerance. Other genes relate to pH response, including those encoding the weak-acid transcription factor War1, Rim101 that mediates the alkaline-pH response, and many of Rim101’s target genes (Obara and Kihara 2014, Obara and Kamura 2020).

We identified a contrasting group of genes whose deletion was actually beneficial to IILs and other cationic compounds (101 genes in Cluster 28, Fig. 5A), and these may also relate to pH stress. This group includes the kinase Ptk2, which regulates the plasma membrane H^+^-ATP pump Pma1, and the kinase Sky1, which has been implicated in ion homeostasis, potentially through regulation of Trk1/2 potassium transporters or other potassium homeostasis components (Goossens et al. 2000, Erez and Kahana 2002, Forment et al. 2002, Eraso et al. 2006). Cluster 28 was heavily enriched for genes localized to the Golgi and vacuole, including the vacuolar V-ATPase Vph1 that maintains the acidic vacuolar pH and Stv1 in the Golgi, which creates a gradient that facilitates the transport of substrates such as Ca^2+^, toxins, and amino acids (Martínez-Muñoz and Kane 2008, Cyert and Philpott 2013). Beneficial deletions were also enriched for genes encoding proteins localized to the endoplasmic reticulum (ER) and plasma membrane—but the group was also enriched for genes identified by Lee et al. 2014 whose deletion causes sensitivity to ergosterol and fatty acid depletion, cell wall damage, and plasma membrane duress (see Fig. 5B and Dataset4_HyperG_cdt (Supporting Information)). Together, the relationships between IIL and cationic dyes suggest that cationic stress impacts ion balance, pH, and the secretory pathway, in an opposing way to published compounds that induce lipid and cell-surface stress (see Discussion).

Despite the similarities with cationic dyes, other distinctions reveal responses unique to the IILs EMIM-Cl and BMIM-Cl. Most striking was genes in Cluster 27, whose deletion was beneficial for growth in CV and NAO but extremely detrimental for surviving IILs and also the iron chelating agent 2,2′-dipyridyl. This group was heavily enriched for genes involved in the biosynthesis of amino acids, including lysine, histidine, and arginine, as well as mitochondrial functions, including the electron transport chain. IILs have been proposed to disrupt the mitochondrial membrane potential and its activity (Dickinson et al. 2016), which could account for the response. The relationship between IILs and 2,2′-dipyridyl with regard to these gene deletions was unexpected. In fact, some genes in Cluster 27 whose deletion is deleterious for IIL tolerance (such as *RIM1, AIM22, IMG2, HMI1, SWS2, LIP2*, and *PCP1*) were among genes identified by Lee et al. as important to manage iron homeostasis or to provide resistance to mitochondrial stressors (*P*-value ≤ 10^−4^) (Fig. 5B). These results raise the possibility that IILs either chelate iron or impact mitochondria in a way that disrupts iron stores.

### Phenolic compounds display antagonistic responses with cationic toxins

Phenolic compounds fell into multiple subclasses in Fig. 3. Clustering the fitness profiles for gene knockouts important for growth in one or more phenolic inhibitors revealed subtle differences that distinguished the phenolic classes (see Fig. S3, Supporting Information). Although there were some functional enrichments for these class-specific responses, the mechanisms for their differential effects remain unclear; nonetheless, these results confirm that different phenolic compounds can impart distinct effects on cells.

Several clusters of gene deletions had broadly similar fitness benefits or defects shared across many of the phenolic compounds; strikingly, many were the same as those discussed above for IIL tolerance, but they had the opposite fitness effects for phenolic compounds. For example, many of the Cluster 28 genes whose deletion was beneficial to IIL tolerance were required for normal growth in multiple phenolic compounds, such as coumaric and ferulic acid. Some of these genes (*ARL1, MAK10, DRS2, YPT6, COG6, COG8*, and *VPS38*) were previously identified as being important for ferulic acid tolerance under aerobic conditions (Fletcher et al. 2019); our results show they are important regardless of oxygen condition. Genes involved in Golgi function, cellular trafficking, membrane, and cell wall stress were important for growth in the presence of phenolics but detrimental in the presence of IILs. In contrast, deletion of genes in Cluster 27 involved in mitochondrial function, amino acid synthesis, iron homoeostasis and mitochondrial stress response were all beneficial to growth in phenolics but detrimental for growth in IILs. So too were genes in Cluster 8, many of which function in pH maintenance and ion homeostasis. Thus, deletions of these genes affect ion and pH homeostasis in opposite ways for tolerance to cationic and phenolic compounds, which may have opposing effects that raise or lower the intracellular pH, respectively (see Discussion).

**Figure 6.**
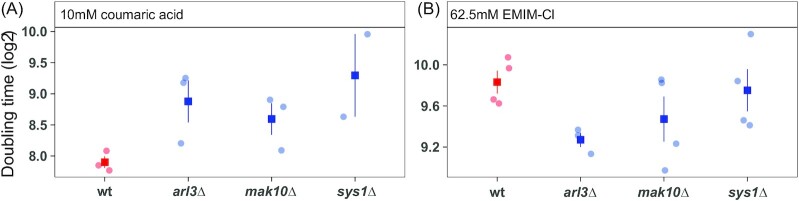
Arl pathway mutants display antagonistic pleiotropy with IILs and phenolic acids. The indicated strains were cultured anaerobically in synthetic hydrolysate medium containing 10 mM *p*-coumaric acid (**A**) or 62.5 mM EMIM-Cl (**B**). Doubling times were estimated from exponential growth rates from at least 3 independent biological replicates.

To validate these antagonistic effects, we measured growth rates in strains lacking several of these genes that function together in cargo trafficking at the trans-Golgi Network (Graham 2004, Yu and Lee 2017). Wild-type, *arl3Δ*, *mak10Δ*, and *sys1Δ* mutant strains were cultured separately in medium containing EMIM-Cl or coumaric acid. The three mutant strains generally grew faster in the IIL EMIM-Cl compared to the wild-type strain and more slowly in coumaric acid, although these differences were not significant due to wide variation in mutant doubling times (Fig. 6). The molecular mechanism of this tradeoff is unclear, but it may relate to the role of Arl1 (and presumably its interacting partners in the *ARL* pathway) in regulating ion influx and membrane potential (Munson et al. 2004). The *ARL* pathway genes may have opposing functions to the ion transporters and regulators in Cluster 8 (see Discussion).

### GVL shares similarities in cell response with several biofuel products

**Figure 7.**
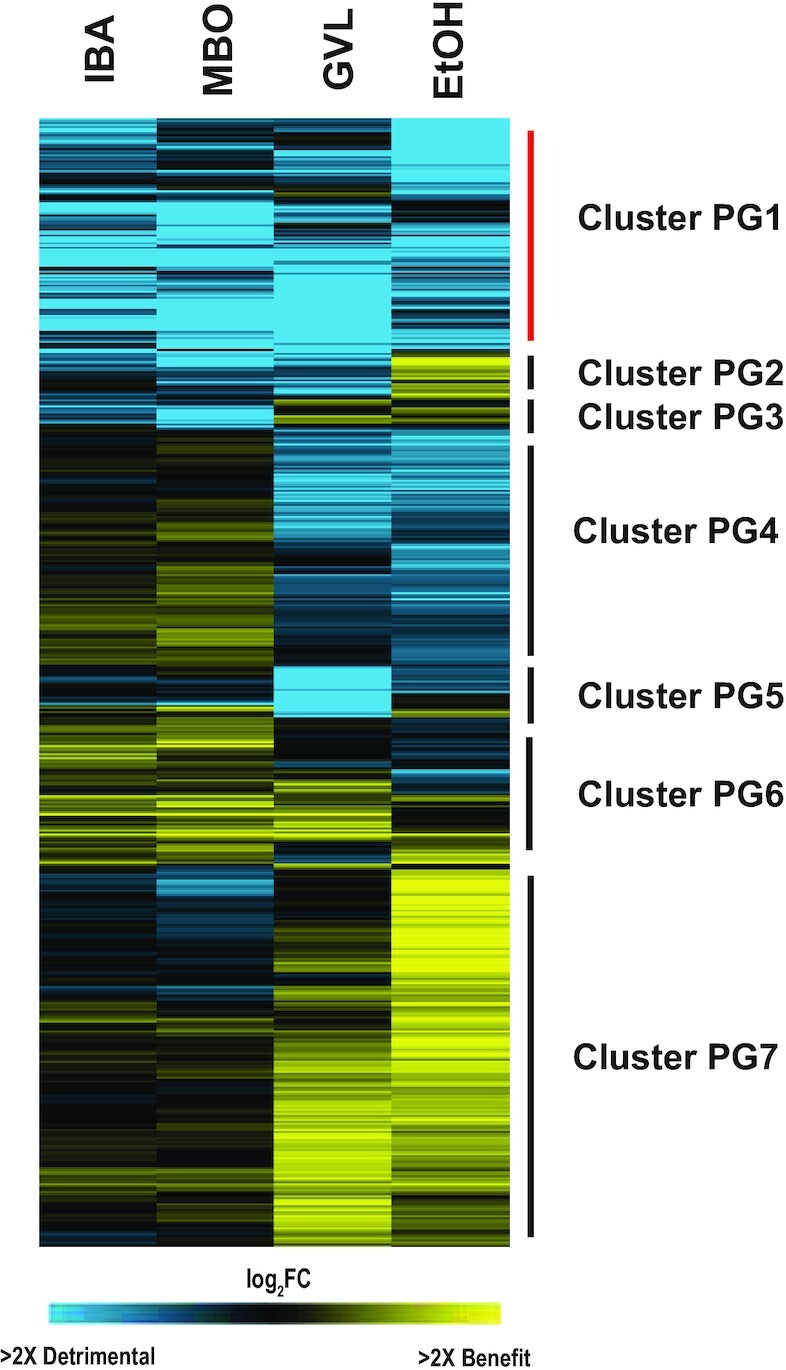
GVL shares similarities with different biofuel products. 2646 gene deletions significant for at least one of the four compounds (IBA, MBO, EtOH, and GVL) were hierarchically clustered and represented in a heatmap as described in Fig. 5. Clusters are indicated next to side bar for responses common (red) or unique (black) to subsets of solvents. Clusters are defined as PG (Product-GVL) to distinguish them from Fig. 5 clusters.

Three compounds with hydrophobic properties, MBO, IBA, and GVL, were grouped together by chemicals classification in Fig. 3. Interestingly, the chemical genomic profile for GVL was also similar to that of ethanol, to a greater extent than the other compounds. To better understand these differences, we selected genes whose deletion impacted fitness upon MBO, IBA, GVL, and ethanol treatment and clustered the genes separately (Fig. 7).

A substantial fraction of 548 genes (Fig. 7, Cluster PG1) were required for survival of all four compounds to varying degrees. Many of these genes are related to inter-organelle communication, including members of the ERMES (*MDM10, MDM34*, and *GEM1*) and GET (*GET1* and *GET2*) complexes that are important for ER and mitochondrial contacts and/or morphology, proteins localized to the vacuole and the peroxisomes, and proteins important for nucleus-mitochondrial signaling (such as *RTG1*, *RTG2*, and *TOR1*). Many proteins implicated in protein transport were important for surviving these solvents (*P* = 2.6e-5, hypergeometric test), as well as proteins involved in monoubiquitination (*P* = 1e-4) that are important for endocytosis. Together, these fitness effects suggest that the solvents perturb protein trafficking and inter-organelle communication. ERMES and GET complexes also influence phospholipid biosynthesis and transfer, as well as targeting and insertion of tail-anchored proteins in the ER membrane (Schuldiner et al. 2008, Zhang et al. 2014, Onishi et al. 2018). Genes related to lipid biosynthesis were also enriched (*P* = 5.3e-6), including those involved in ergosterol and fatty acid biosynthesis (*ERG2, ERG3, SUR4, TYR1*, and *ARO2*), as well as genes involved in tryptophan biosynthesis (*TRP2, TRP3*, and *TRP4)*, which have been also observed to function in isobutanol tolerance (Kuroda et al. 2019). Alcohols, as well as GVL, are known to permeabilize membranes, which may contribute to these effects (Ding et al. 2009, Huffer et al. 2011). Interestingly, many of the gene deletions detrimental for growth in these four compounds fell into Cluster 30 from Fig. 5A, revealing that the deletions produce fitness benefits to IILs and other cationic compounds. These genes were significantly enriched in functions for protein urmylation (*P* = 0.0092), a ubiquitin-like pathway involved in nutrient sensing and budding (Furukawa et al. 2000, Goehring et al. 2003).

Interestingly, genes required for tolerating the four solvents also included many genes involved in cell cycle progression, mainly G2/M transition of mitotic cell cycle (*PIN4, CLB3, HSL1, BIK1, KIP2, KCC4*) and encode proteins of the spindle pole body and/or microtubules, the septin ring, or the bud neck (*KCC4, BIK1, BIM1, SSD1, BUD6, ELM1*). Figure 5B revealed some similarity in functional enrichments between these compounds and benomyl, a known tubulin depolymerizer that disrupts cell cycle progress, since genes annotated as ‘microtubule’ were enriched among genes required to tolerate MBO, IBA, GVL, and to a lesser extent ethanol.

Clustering the gene-fitness profiles for the four chemicals revealed similarities between GVL and ethanol that were not seen for MBO and IBA. Nearly 500 gene deletions were detrimental to growth in the presence of GVL and ethanol (Fig. 7, Cluster PG4), and this gene list was enriched for carbohydrate metabolism and glycosphingolipid synthesis genes (*P *< 0.001, Hypergeometric test). In contrast, other gene deletions were beneficial to GVL and ethanol stress compared to IBA and MBO (Fig. 7, Cluster PG7); many of these overlapped with Cluster 8 from Fig. 5A, whose deletion presented a fitness defect in response to cationic compounds and implicated the importance of pH balance (see Discussion). Although results in this analysis suggest a partly shared mechanism among these four compounds, we also observed differences in the cellular adaptive response to them, especially between IBA and ethanol. This opposite effect of the two main products may be of importance when developing tolerance approaches according to the compound generated.

## Discussion

Chemical genomics has been successfully applied in previous biofuel studies, helping to uncover new insights that foster microbial engineering for increased efficiency. Many prior studies used such approaches to investigate cellular defense mechanisms to hydrolysates or to specific compounds studied in isolation and under aerobic conditions (Pereira et al. 2014, Dickinson et al. 2016, Ong et al. 2016, Bottoms et al. 2018, Fletcher et al. 2019). Here, we undertook a broader, comparative approach to study a variety of lignocellulose-derived inhibitors, solvents, and biofuel products relevant to multiple pretreatment methods and under anaerobic conditions. We used a drug-sensitive strain library that allows to uncover a large number of genes involved in the adaptive response, and consequently, to reveal specific mechanisms of toxicity of the toxins studied. Despite the high sensitivity of this strain background, our validation experiments with a less sensitive strain confirm findings under the conditions used here. However, we note that strains can vary significantly in their tolerance profiles due to genetic background; understanding the influence of genetic background on toxin tolerance is an important and active area of research (Sardi and Gasch 2017, 2018, Cámara et al. 2022). We used concentrations of each inhibitor for a consistent growth among them, but differences in composition of inhibitors in hydrolysates from batch to batch could cause a variance in the severity of the stress for the same inhibitors. This approach allows for both a deeper understanding of defense mechanisms while developing a rich dataset for further analysis, in certain industrially relevant fermentative conditions.

Our results have important implications on both cellular defense and engineering strategies. First, comparative analysis of a larger set of inhibitors helped to distinguish shared and unique mechanisms of stress. Clustering gene fitness contributions across many inhibitors helped to distinguish subsets of genes with shared, or opposing, effects across compounds including those with known mechanisms. For example, although the molecular details remain to be worked out, the shared responses suggested that maintenance of the plasma membrane potential as well as secretion, membrane/cell surface stress responses, pH, mitochondrial function, and lipid biosynthesis play important roles in IIL and cation tolerance.

However, many of these responses were inversely important for surviving phenolic compounds, suggesting that these inhibitor classes are provoking opposing effects to the same cellular physiology. We propose that a key aspect of that physiology is influenced by ion and pH homeostasis, in opposing ways for tolerance to cationic compounds, which may induce alkalinization, and some phenolic compounds that acidify the cell. Cation influx is predicted to raise internal pH due to corresponding efflux of H^+^, and the requirement of the Rim101 alkaline-response regulon for tolerating these compounds is consistent with this notion (Ariño et al. 2010). IILs can insert into lipid bilayers, and permeability of IIL can be affected by differences in lipid composition, fluidity, and other properties (Gal et al. 2012, Cook et al. 2019). Perturbations to lipid bilayer asymmetry can directly activate Rim101, underscoring the intimate relationship between membrane status and pH (Ikeda et al. 2008, Obara and Kamura 2020). The inverse relationships extended to gene deletions sensitized to phospholipid flipase perturbation, including Neo1; ergosterol biosynthesis; and vesicle trafficking, all of which also influence vacuolar pH homeostasis and membrane biology (Brett et al. 2011).

In contrast, phenolic compounds (especially phenolic acids) and other stresses may decrease cellular pH leading to acid stress. Many of these compounds may become deprotonated inside the cell, in a similar manner to other organic acids. Ethanol stress is known to acidify the cytosol due to plasma membrane permeabilization and H^+^ influx (Madeira et al. 2010, Lam et al. 2014, Charoenbhakdi et al. 2016), thereby producing inverse dependencies on pH-related genes.

The widespread antagonistic effects for certain mutants exposed to different classes of inhibitors raise some considerations for strain engineering. While previous strategies have successfully identified engineering strategies that improve tolerance of single inhibitors (Dickinson et al. 2016, Bottoms et al. 2018, Higgins et al. 2018, Fletcher et al. 2019, Kuroda et al. 2019), our results show the importance of considering that those genetic changes could affect resistance to other toxins in the same hydrolysates, producing a strain that is, in the end, less fit for the complex mixture. Hydrolysate composition is influenced by different factors such as the type of biomass, plant growth conditions, or pre-treatment methods (Cunha et al. 2019). Phenolics are produced in higher concentrations in AFEX-treated biomass than acid hydrolysates, including specific phenolic compounds, such as amides (Keating et al. 2014). Our results showed some differences between amides and ketones when compared to other phenolics, which would also imply variable responses according to the pre-treatment method. The antagonistic analysis between couples of inhibitors also showed the importance of selecting the appropriate pre-treatment according to the desired product to decrease negative effects during the fermentation. An alternative strategy may be to engineer suites of strains with resistance to sets of inhibitors that co-fluctuate across hydrolysate types or batches, and to choose the best strain for each batch of hydrolysate based on their composition and the desired product.

## Conclusion

We anticipate that the rich dataset of yeast cellular responses to hydrolysate toxins presented here will aid in future biofuel studies including strain engineering. Selecting gene targets may be most effective by considering the response of engineered strains like the gene-deletion lines studied here to multiple toxins, including those that may be present in the same hydrolysate. This data set may also provide a useful backdrop for modeling toxins in complex mixtures, by comparing chemical genomic footprints in those mixtures to the single inhibitors studied here.

## Author's contributions

Conceived and designed the study: APG, TKS, CTH. Performed chemical genomic experiment and validations: LL, DJD, MKMY. Performed computational analysis: MP, EVP, KJF. Analyzed the data: EVP, KJF, APG, TKS. Wrote the paper: EVP, KJF, APG, TKS, with input from the other authors. All authors read and approved the final manuscript.

## Supplementary Material

foac036_Supplemental_Files

## Data Availability

All sequencing data are available in the NIH GEO database under accession number GSE186866. The data sets used and/or analyzed during this study are included within this article and its supplementary information files.
