# Supplementary material for: Comparative chemical genomic profiling across plant-based hydrolysate toxins reveals widespread antagonism in fitness contributions
Source: FEMS Yeast Res. 2022 Jul 26;22(1):foac036. doi: 10.1093/femsyr/foac036 (PMC9508847; doi:10.1093/femsyr/foac036)
Supplement: foac036_Supplemental_Files [file foac036_supplemental_files.zip › Dataset_Legends.docx]

**Dataset1_HyperG.** Dataset containing functional enrichment for each compound calculated using a Hypergeometric test. Each row is functional categories, and each column is an inhibitor. Values are shown as -log_10_(p-value) (see Methods).

**Dataset2_mclust_cdt.** Dataset containing clustered data from mclust analysis shown in Figure 5A. Rows define genes, and columns are inhibitors. Fitness is shown as log2 values (see Methods).

**Dataset3_HyperG_mclust.** Dataset containing functional enrichments for clusters from Dataset2 shown in Figure 5A, using a hypergeometric test (see Methods).

**Dataset4_HyperG_cdt.** Dataset containing the hierarchical clustering of functional categories shown in Figure 5B. Rows define categories, and columns are inhibitors. Values are shown as -log10(p-value) as described in the text (also, see Methods).
