# Supplementary material for: Comparative chemical genomic profiling across plant-based hydrolysate toxins reveals widespread antagonism in fitness contributions
Source: FEMS Yeast Res. 2022 Jul 26;22(1):foac036. doi: 10.1093/femsyr/foac036 (PMC9508847; doi:10.1093/femsyr/foac036)
Supplement: foac036_Supplemental_Files [file foac036_supplemental_files.zip › Figure_S1.pdf]

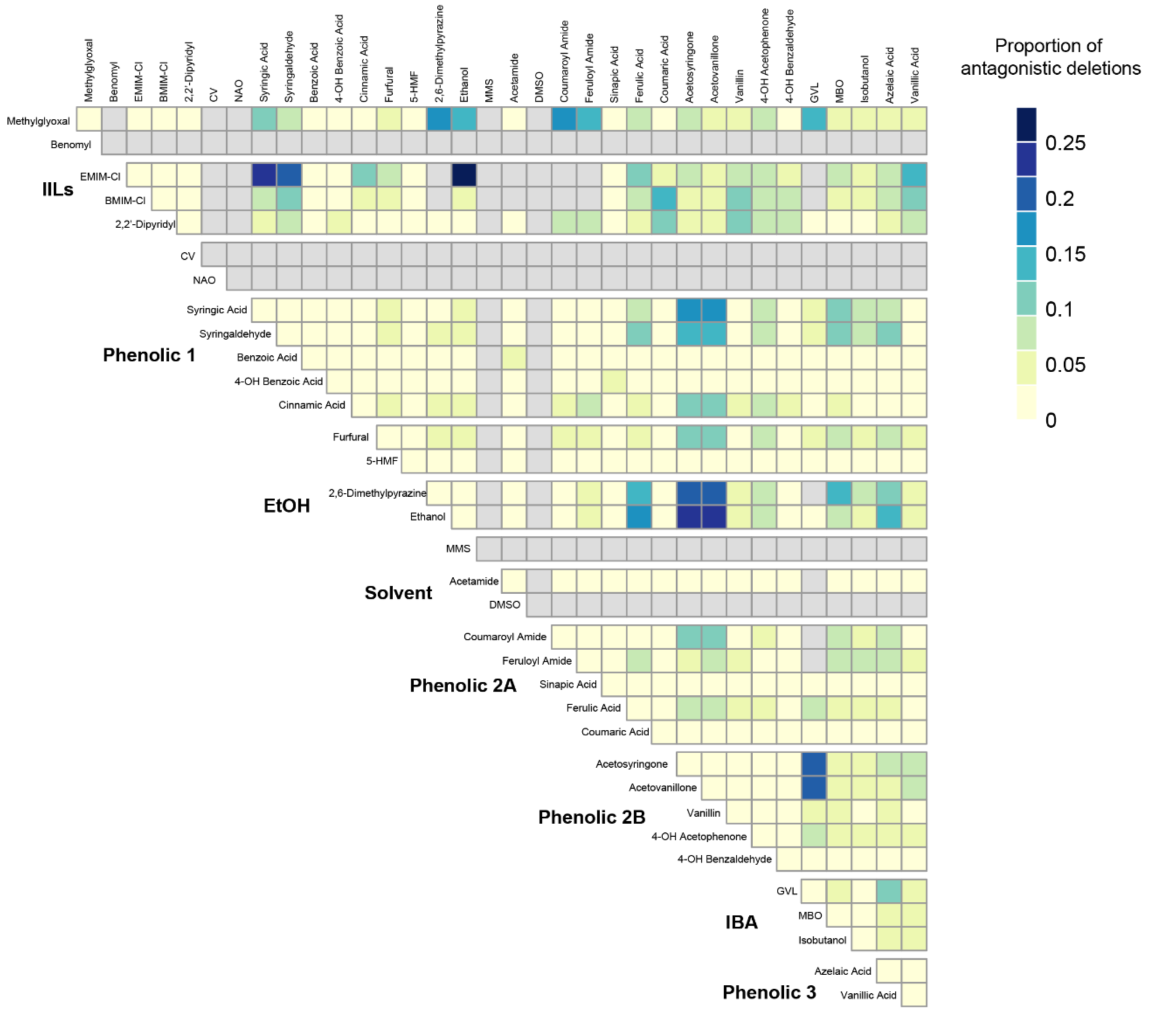

Figure S1. Heatmap showing the proportion of antagonistic deletions in pairwise unions of significant deletions. Rows and columns are ordered according to the inhibitor groupings assigned by hierarchical clustering of Pearson coefficients in Figure 3. Pairwise combinations that would not co-occur in real hydrolysates were not calculated.
