# Supplementary material for: Comparative chemical genomic profiling across plant-based hydrolysate toxins reveals widespread antagonism in fitness contributions
Source: FEMS Yeast Res. 2022 Jul 26;22(1):foac036. doi: 10.1093/femsyr/foac036 (PMC9508847; doi:10.1093/femsyr/foac036)
Supplement: foac036_Supplemental_Files [file foac036_supplemental_files.zip › Figure_S2.pdf]

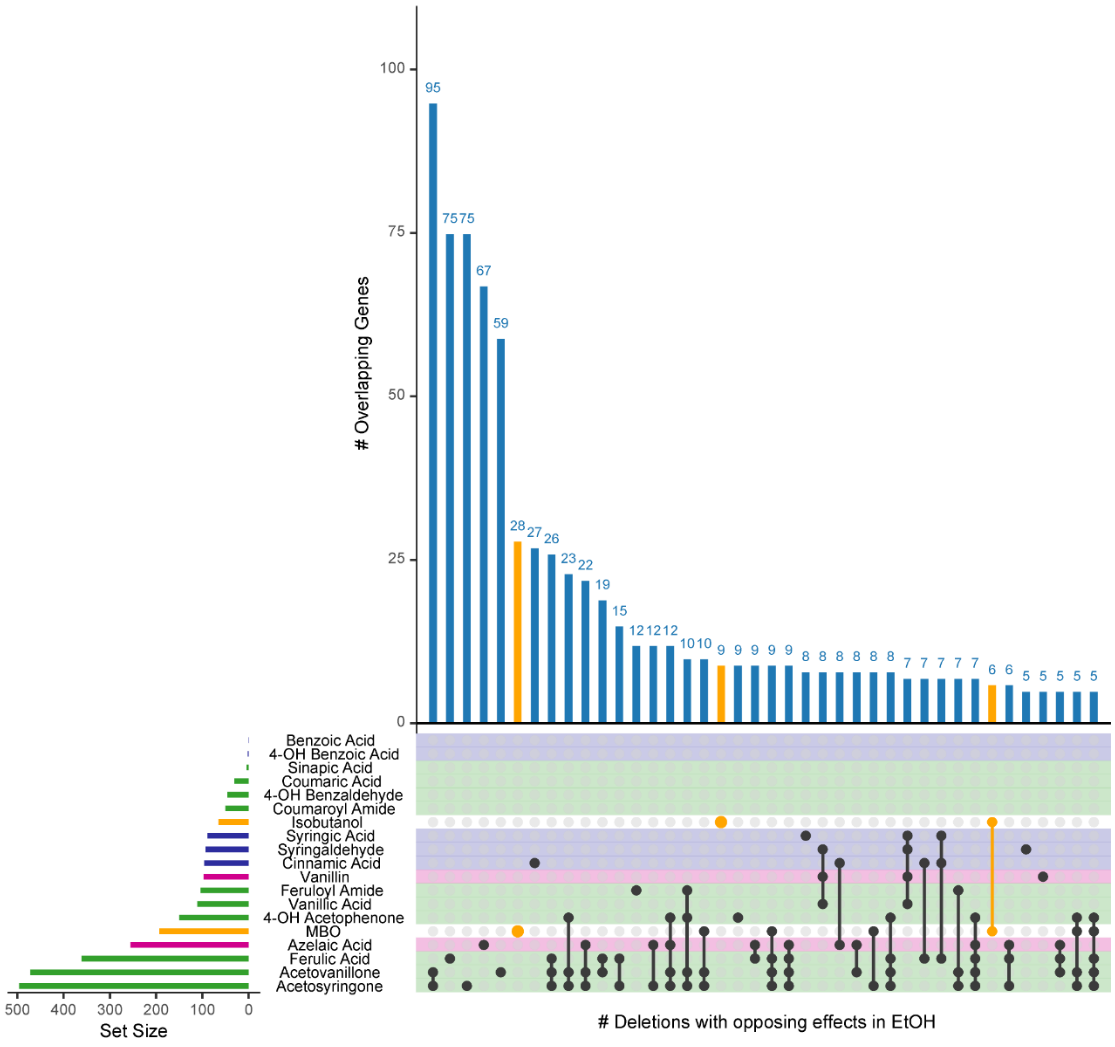


**Figure S2. UpSet plots showing the intersection of significant deletions that have opposing fitness effects in ethanol and phenolic inhibitors.** Bar plots to the left of inhibitor names show the Set Size of all antagonistic genes in the specific pairwise comparison. Solid dots indicate intersections, and the vertical bar plot shows intersection size. Colors of inhibitor set size bar plots and intersection rows correspond to inhibitor groupings assigned by hierarchical clustering of Pearson coefficients in Figure 3 (blue – benzoic, green – phenolic 2A&B, magenta – phenolic 3). Biofuels are indicated with orange bars. Most antagonism between ethanol and other inhibitors is unique. The shared basis of antagonism between ethanol and other biofuels (6 genes, orange) is smaller than unique antagonistic deletions (orange).
