# Supplementary material for: Comparative chemical genomic profiling across plant-based hydrolysate toxins reveals widespread antagonism in fitness contributions
Source: FEMS Yeast Res. 2022 Jul 26;22(1):foac036. doi: 10.1093/femsyr/foac036 (PMC9508847; doi:10.1093/femsyr/foac036)
Supplement: foac036_Supplemental_Files [file foac036_supplemental_files.zip › Figure_S3.pdf]

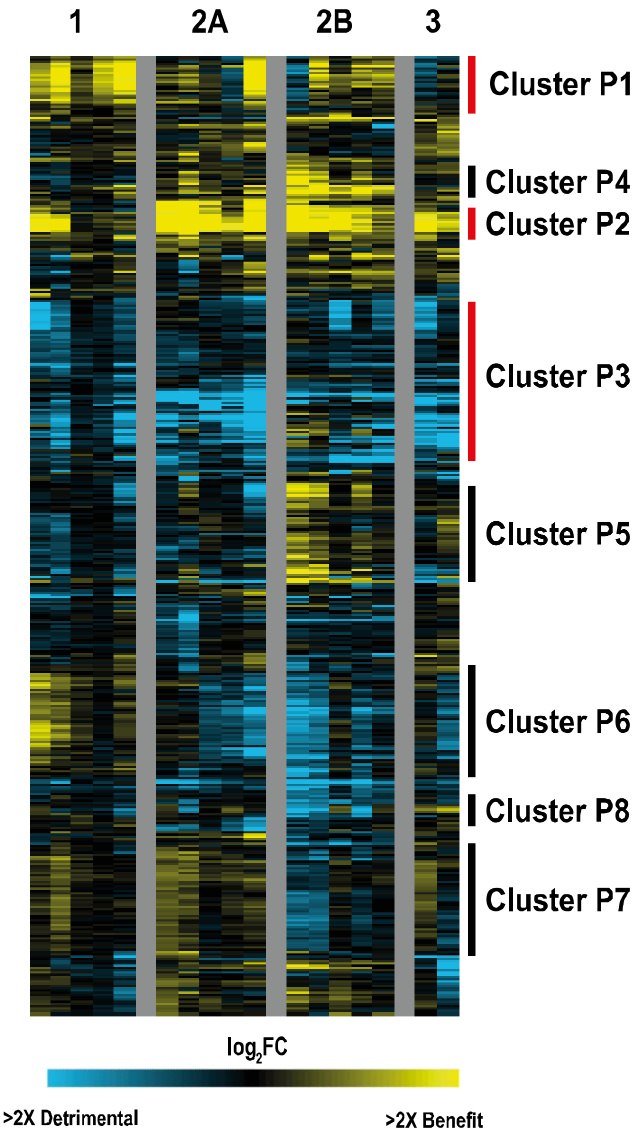

Figure S3. Phenolic classes are differentiated by specific gene groups. 2621 genes whose deletion impacts fitness for at least one phenolic compound (FDR<0.05) were organized by hierarchically clustering and represented in a heatmap as the $\log_2$ of the inhibitor/compound ratio and defined as fitness defect (FD) in blue or fitness benefit (FB) in yellow. Specific subclusters that differentiate the phenolic compounds are indicated to the right of the heat map next to the side bar for mostly common (red) and different (black) effects across the classes.
