# Supplementary material for: Comparative chemical genomic profiling across plant-based hydrolysate toxins reveals widespread antagonism in fitness contributions
Source: FEMS Yeast Res. 2022 Jul 26;22(1):foac036. doi: 10.1093/femsyr/foac036 (PMC9508847; doi:10.1093/femsyr/foac036)
Supplement: foac036_Supplemental_Files [file foac036_supplemental_files.zip › Table_S1.pdf]

Table S1. **Chemicals and concentrations used in chemical genomic studies** (see Methods, “Chemical genomic experiment”).

Chemical Additive	IC_{30} Concentration	Dissolved in DMSO?	CAS Number or reference	Vendor/Source	Catalog #
**IONIC LIQUIDS**					
EMIM-Cl / [C_2C_{1i}m]Cl	50 mM	No	65039-09-0	Sigma-Aldrich	272841
BMIM-Cl / [C_4C_1im]Cl	8 mM	No	79917-90-1	Sigma-Aldrich	94128
**CATIONIC DYES**					
Crystal Violet (CV)	15 uM	No	548-62-9	Fisher Scientific	C8126
Nonyl-acridine orange (NAO)	5 uM	No	75168-11-5	Sigma-Aldrich	A7847
**END-PRODUCTS**					
2-Methyl-3-buten-2-ol (MBO)	1.50%	No	115-18-4	Sigma-Aldrich	136816
Ethanol	4%	No	64-17-5	Various	
Isobutanol (IBA)	0.75%	No	78-83-1	Acros Organics	41265
**FURANS**					
5-OH Methylfurfural (5-HMF)	3.3 mM	No	67-47-0	Acros Organics	121460050
Furfural	8 mM	No	98-01-1	Fisher Scientific	F94-500
**PHENOLIC COMPOUNDS**					
4-OH Acetophenone	5 mM	Yes	99-93-4	Sigma-Aldrich	278564
4-OH Benzaldehyde	4 mM	Yes	123-08-0	Sigma-Aldrich	144088
4-OH Benzoic Acid	6 mM	Yes	99-96-7	Sigma-Aldrich	240141

Acetosyringone	5 mM	Yes	2478-38-8	Sigma-Aldrich	D134406
Acetovanillone	3 mM	Yes	498-02-2	Sigma-Aldrich	W508454
Benzoic Acid	0.12 mM	Yes	65-85-0	Sigma-Aldrich	242381
Caffeic Acid	7.5 mM	Yes	331-39-5	Sigma-Aldrich	C0625
Cinnamic Acid	0.38 mM	Yes	140-10-3	Sigma-Aldrich	C80857
Coumaroyl Amide	3 mM	Yes	Keating et al., 2014	Y. Zhang	
Ferulic Acid	0.94 mM	Yes	537-98-4	Sigma-Aldrich	W518301
Feruloyl Amide	2 mM	Yes	Keating et al., 2014	Y. Zhang	
p-Coumaric Acid	3.3 mM	Yes	501-98-4	Sigma-Aldrich	C9008
Sinapic Acid	1.5 mM	Yes	530-59-6	Sigma-Aldrich	D7927
Syringaldehyde	2.2 mM	Yes	134-96-3	Sigma-Aldrich	S1602
Syringic Acid	15 mM	Yes	530-57-4	Sigma-Aldrich	S6881
Vanillic Acid	6.7 mM	Yes	121-34-6	Sigma-Aldrich	H36001
Vanillin	5 mM	Yes	121-33-5	Sigma-Aldrich	V1104
**SOLVENTS**					
DMSO	2.50%	No	67-68-5	Sigma-Aldrich	D-8779
Gamma valerolactone (GVL)	1.5%	No	108-29-2	Acros Organics	140795000
**OTHER COMPOUNDS**					
Azelaic Acid	10 mM	Yes	123-99-9	Sigma-Aldrich	246379
2,2'-Dipyridyl	18 ug/mL	No	366-18-7	Acros Organics	117500100

Benomyl	10 ug/mL	No	17804-35-2	NA	NA
Methylmethane sulphonate (MMS)	0.01%	No	66-27-3	Sigma-Aldrich	129925
Acetamide	250 mM	No	60-35-5	Sigma-Aldrich	00160
Methylglyoxal	7.5 mM	No	78-98-8	MP Biomedicals	155558
2,6-Dimethylpyrazine	38 mM	No	108-50-9	Sigma-Aldrich	W327301
2,4-Dimethylimidazole	6 mM	No	930-62-1	Alfa Aesar	A11949
Levulinic Acid	10 mM	No	123-76-2	Alfa Aesar	A10813
Mycobutanil	13 ug/mL	No	88671-89-0	Sigma-Aldrich	34360
QUADRIS-1	0.08%	No	119446-68-3	Syngenta	
QUADRIS-2	0.04%	No	119446-68-3	Syngenta	
Sodium Acetate	150 mM	No	127-09-3	Fisher Scientific	S8750
Sodium Butyrate	0.19 mM	No	156-54-7	Fisher Scientific	S1999
Sodium Glyoxylate	0.19 mM	No	918149-31-2	Sigma-Aldrich	G4502
2-Methylimidazole	3.8 mM	Yes	693-98-1	Sigma-Aldrich	M50850
4(5)-Methylimidazole	3.3 mM	Yes	822-36-6	Sigma-Aldrich	199885

Keating et al., 2014 10.3389/fmicb.2014.00402
